# KLF4-mediated upregulation of CD9 and CD81 suppresses hepatocellular carcinoma development via JNK signaling

**DOI:** 10.1038/s41419-020-2479-z

**Published:** 2020-04-29

**Authors:** Yandong Li, Shijun Yu, Li Li, Jingde Chen, Ming Quan, Qinchuan Li, Yong Gao

**Affiliations:** 1 Department of Oncology, Shanghai East Hospital, Tongji University School of Medicine, Shanghai, 200120 China; 2 Department of Thoracic Surgery, Shanghai East Hospital, Tongji University School of Medicine, Shanghai, 200120 China

**Keywords:** Oncogenes, Cell growth

## Abstract

Tetraspanins CD9 and CD81 frequently serve as the surface markers of exosomes, which are involved in intercellular communication during tumor progression. KLF4 is a well-known tumor suppressor in various cancers. This study aims to investigate the relationship between KLF4 and CD9/CD81 in hepatocellular carcinoma (HCC). The results showed that CD9 and CD81 were transcriptionally activated by KLF4 in HCC cell lines. Decreased expressions of CD9 and CD81 were found in most HCC tumor tissues and predicted advanced stages. Furthermore, KLF4 expression was positively associated with CD9 and CD81 expression in HCC specimens. Functionally, overexpression of CD9 and CD81 inhibited HCC cell proliferation in vitro and in vivo and silencing CD9 and CD81 displayed opposite phenotypes. Mechanistically, we found that JNK signaling pathway may be involved in the growth suppression mediated by CD9 and CD81. In addition, increased expression of KLF4, CD9 or CD81 had no obvious impact on exosome secretion from HCC cells. Collectively, we identified CD9 and CD81 as new transcriptional targets of KLF4 and the dysregulated KLF4-CD9/CD81-JNK signaling contributes to HCC development. Our findings will provide new promising targets against this disease.

## Introduction

Exosome, a major component of extracellular vesicles (EVs), is a nanometer-sized membrane structure ranging in diameter from 30 to 150 nm, which could be secreted by most cell types^1^. A number of reports have demonstrated that exosomes participate in cell–cell communications in both physiological activities and pathological changes by transferring differential cargos, including proteins, DNAs, microRNAs, lncRNAs, and mRNAs^2–4^. In cancer, exosome-mediated transfer has been shown to contribute to tumor cell proliferation, metastasis, and signal transduction^5–7^. Cancer cells also secrete more exosomes than normal tissue cells, which raises the possibility that exosomes can serve as potential diagnostic markers for cancer patients^8–10^.

Besides the contents within exosomes, the surface molecules are also involved in tumorigenesis^11,12^. Exosomes are commonly characterized by unique surface proteins including the tetraspanins (CD9, CD63, CD81, etc.) and other molecules (intergrins, Alix, Tsg101, etc.)^13–15^. The tetraspanins CD9 and CD151 have been reported to enhance the migration and invasion abilities of prostate cells, which suggest that exosomal tetraspanins might act as a driver of metastasis in prostate cancer^16^. The study by Logozzi et al. proved that CD63-postive exosomes are significantly higher in melanoma patients comparing with healthy people^17^. However in another report, CD63 has been shown to act as a negative driver of epithelial-to-mesenchymal transition (EMT) in the same cancer type^18^. To date, little is known about the expression and significance of surface molecules of exosomes in most cancer types, and the molecular mechanisms by which they regulate cancer development have not been fully understood.

Krüppel-like factor 4 (KLF4) is a zinc-finger transcription factor from KLFs family that shares a C-terminal three-zinc-finger DNA-binding domain^19,20^. KLF4 is usually found to be expressed in post-mitotic and terminally differentiated epithelial cells in the skin, lungs and gastrointestinal tracts and acts as a significant regulator of cell activities such as proliferation, differentiation, and apoptosis^19,21–23^. Mounting evidence suggests that KLF4 plays a critical role in the progression of various cancers^24,25^. It has been implicated that KLF4 inhibits cell proliferation and tumor growth in neuroblastoma^26^, pancreatic^27^, gastric^24^, colorectal^28^, hepatocellular^29^, and lung carcinomas^30^. On the other hand, KLF4 is required for maintaining cancer stem cell populations in breast and colon cancer cells^31,32^, and KLF4α, an alternative spliced KLF4 isoform, was identified to act as an oncogene in pancreatic cancer^33^. Although these findings seem to be ambivalent, they implicate an essential role of KLF4 in human malignancies and highlight the necessity to further explore the functions and underlying molecular mechanisms of KLF4 in tumorigenesis.

In the present work, we aimed to investigate whether KLF4 regulates the expression of exosomal surface proteins and their roles in human hepatocellular carcinoma (HCC). We finally identified tetraspanins CD9 and CD81 as new transcriptional targets of KLF4. Reduced expression of CD9 and CD81 was closely associated with low KLF4 expression in HCC tissues. Furthermore, our in vitro and in vivo experiments demonstrated that CD9 and CD81 play important roles in suppressing cell proliferation via negatively regulating MAPK/JNK signaling pathway. These findings suggest that targeting KLF4-CD9/CD81-JNK signaling may be a feasible rationale to develop novel therapeutic strategies against HCC.

## Results

### KLF4 transcriptionally activates CD9 and CD81 expression in HCC cells

**Fig. 1:**
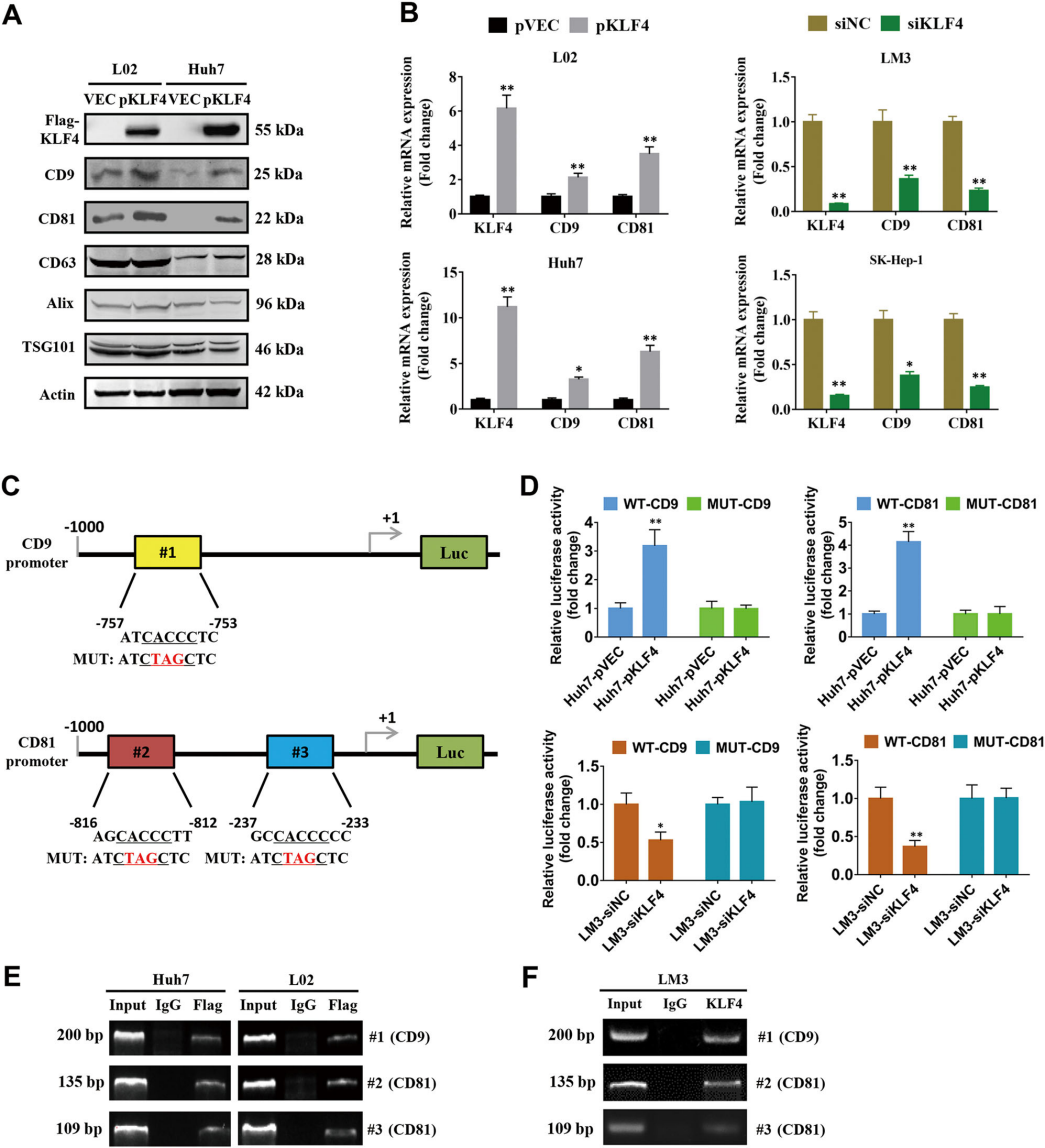
KLF4 positively regulates CD9 and CD81 expression in HCC cells. **a** L02 and Huh7 cells were transiently transfected with KLF4-expression plasmids, then several exosome biomarkers were determined by western blots. **b** The regulation of CD9 and CD81 expression by KLF4 was verified by qPCR analysis after KLF4 overexpression or knockdown. **c** Putative KLF4 binding sites were shown in CD9 and CD81 promoters. **d** Wild type or Mutant CD9/CD81 promoter reporters together with KLF4 overexpression plasmid or siKLF4 were transfected into Huh7 or HCC-LM3 cells as indicated, respectively. After 24 h post transfection, the promoter activities were examined by a dual luciferase assay kit. **e** ChIP assays were performed using chromatins isolated from Huh7 cells transfected with Flag-tagged KLF4 plasmids. **f** ChIP assays were performed using endogenous KLF4 antibody in HCC-LM3 cells. One percent of the total lysates were subjected to PCR amplification before immunoprecipitation and used as Input control, and normal IgG was used as a negative control. *P < 0.05, **P < 0.05.

The specific surface proteins such as CD9, CD63, CD81, Alix, and TSG101 are usually used as one of approaches for identification of collected exosomes from cultured cells, sera, plasma, and tissues in many studies. To elucidate whether KLF4 has a link with these exosomal surface markers, we firstly examined the expression of these proteins after overexpression of KLF4 in HCC cell lines. The western blot results indicated that KLF4 overexpression significantly enhanced CD9 and CD81 expression in L02 and Huh7 cells, while no obvious change were observed in the levels of CD63, Alix, and TSG101 (Fig. 1a). Consistently, the results from qPCR analyses also indicated that KLF4 promoted CD9 and CD81 expression in mRNA level (Fig. 1b). To identify the mechanism by which KLF4 regulates CD9 and CD81 expression, we analyzed the promoter sequences of CD9 and CD81 for the potential KLF4 binding elements 5′-CACCC-3′. As expected, one and two putative KLF4-binding sites were found in the CD9 (#1) and CD81 (#2 and #3) promoter regions, respectively (Fig. 1c). We then generated four luciferase reporters for CD9 (WT-CD9 and MUT-CD9) and for CD81 (WT-CD81 and MUT-CD81). Dual luciferase assays demonstrated that overexpression of KLF4 in Huh7 cells strongly enhanced WT-CD9/CD81 promoter activity, whereas knockdown of KLF4 resulted in opposite effects in HCC-LM3 cells. Both overexpression and knockdown of KLF4 had no significant impact on MUT-CD9/CD81 promoter activity (Fig. 1d). In addition, ChIP assays using prepared chromatins from Huh7 and L02 cells with KLF4 overexpression were conducted. The results revealed that exogenous FLAG-tagged KLF4 protein but not control IgG, could bind directly to the indicated sites of CD9 and CD81 promoter regions (Fig. 1e). Similar results were observed in HCC-LM3 cells with endogenous KLF4 antibody (Fig. 1f). These data strongly suggested that KLF4 has a positive association with the expression of exosomal surface markers CD9 and CD81 in HCC cells.

### CD9 and CD81 are downregulated in HCC samples

**Fig. 2:**
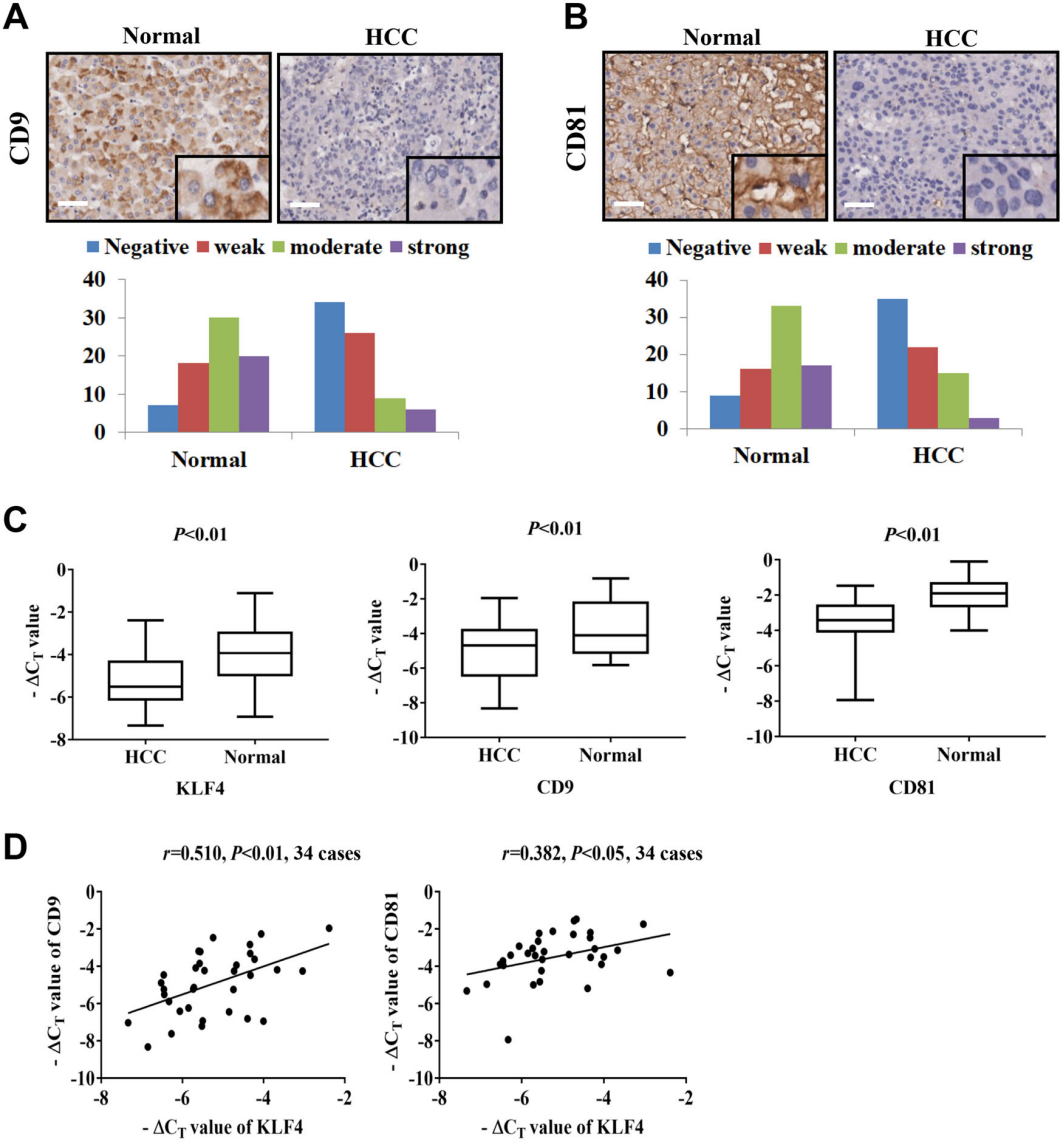
The expression pattern and clinical significance of CD9 and CD81 in HCC. **a**, **b** Representative images of CD9 and CD81 staining in HCC tumor tissues and adjacent normal tissues were shown. Magnification: ×100 and ×400. Scale bar = 50 μm. Column graphs indicate significantly lower expression of CD9 and CD81 in HCC tumor tissues than that in adjacent normal tissues. **c** qPCR analyses indicating lower KLF4, CD9 and CD81 expression in tumor tissues as compared to adjacent normal tissues were shown. **d** The assessment of correlation between KLF4 and CD9 or CD81 expression in HCC tissues using the method of Pearson correlation analysis (*n* = 34).

To investigate the clinical significance of CD9 and CD81 expression in HCC, two tissue microarrays containing 75 paired HCC tissues and adjacent normal tissues were analyzed using specific antibodies against CD9 or CD81 via immunohistochemical staining (IHC). As presented in Fig. 2a and b, CD9 and CD81 expression were frequently downregulated in HCC tissues compared with adjacent normal tissues. Furthermore, their expression levels in stage I/II were significantly higher than in stage III/IV, indicating that both CD9 and CD81 expression were negatively associated with disease stage of HCC (Tables 1 and 2, *P* < 0.05). Similarly, KLF4 expression was also found to be decreased in tumor tissue in the HCC tissue microarray and its low expression predicted advanced progression (Supplementary Fig. 1 and Supplementary Table 1). To explore the expression correlation of CD9 and CD81 with KLF4, qPCR analyses were performed using additional 34 paired HCC tissues and adjacent normal tissues collected from patients with HCC. In agreement with previous reports, KLF4 mRNA expression was remarkably downregulated in tumor tissues, and lower expression of CD9 and CD81 mRNAs were observed in tumor tissues as compared with adjacent normal tissues (Fig. 2c). Further statistical analysis showed that KLF4 expression was positively correlated with the expression of CD9 (*r* = 0.510, *P* < 0.01) and CD81 (*r* = 0.382, *P* < 0.05) in tumor tissues (Fig. 2d). Thus, the findings mentioned above further confirmed the regulation of CD9/CD81 by KLF4 and their correlation with HCC progression.

**Table 1:** The correlation of CD9 expression with clinicopathological parameters of HCC patients.

	Number of cases	Negative, weak	Moderate, strong	*P* value
Age (years)				
> 60	18	14	4	>0.05
≤60	57	46	11	
Gender				
Male	62	50	12	>0.05
Female	13	10	3	
Grading				
I,II	64	50	14	>0.05
III,IV	11	10	1	
Metastasis				
M0	71	58	13	>0.05
M1	4	2	2	
TNM Stage				
I,II	45	32	13	0.018
III, IV	30	28	2

**Table 2:** The correlation of CD81 expression with clinicopathological parameters of HCC patients.

	Number of cases	Negative, weak	Moderate, strong	*P* value
Age (years)				
> 60	18	14	4	>0.05
≤60	57	43	14	
Gender				
Male	62	48	14	>0.05
Female	13	9	4	
Grading				
I,II	64	49	15	>0.05
III,IV	11	8	3	
Metastasis				
M0	71	55	16	>0.05
M1	4	2	2	
TNM Stage				
I,II	45	30	15	0.020
III, IV	30	27	3	

### Overexpression of CD9 and CD81 inhibits HCC cell proliferation

**Fig. 3:**
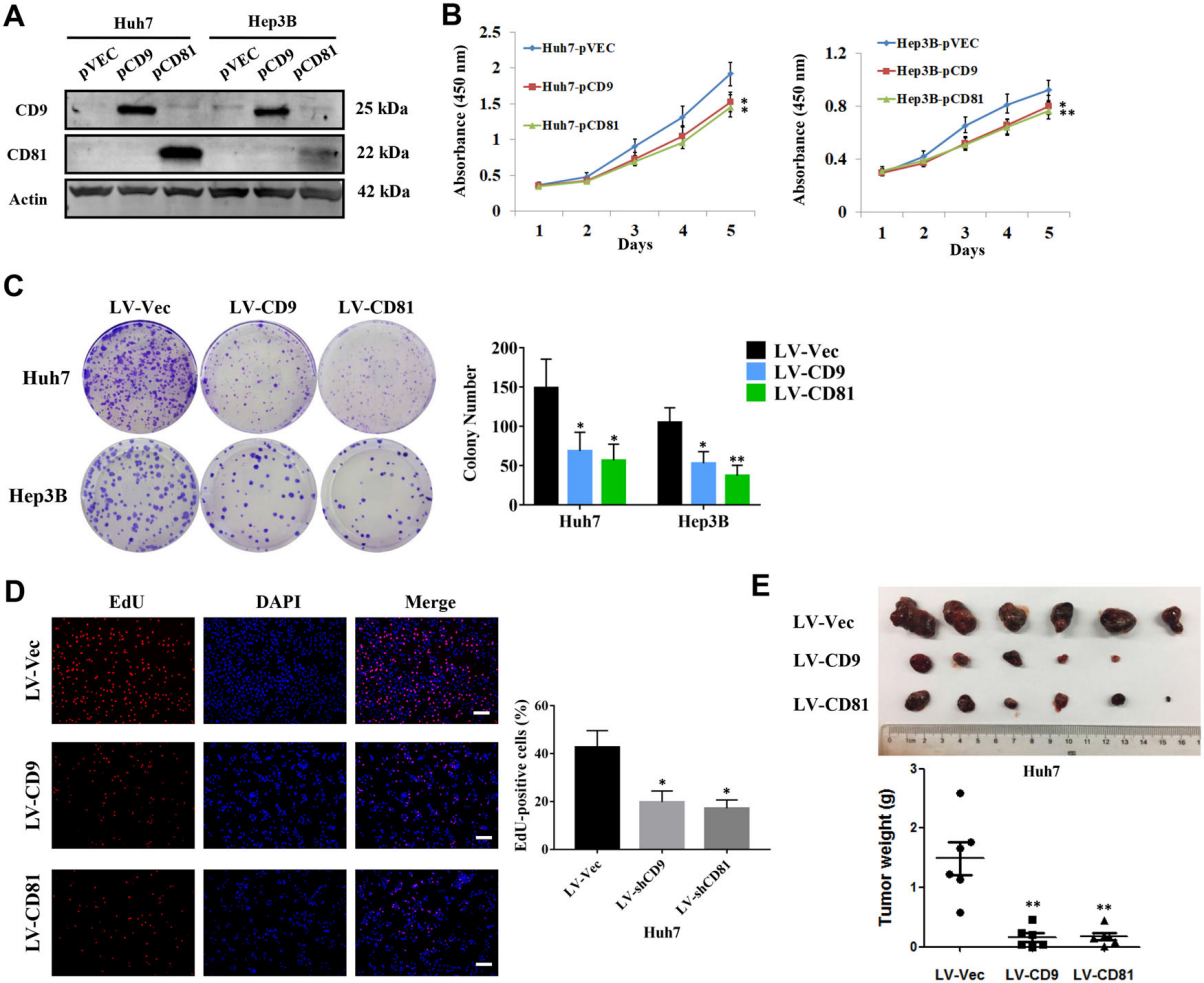
CD9 and CD81 inhibit HCC cell proliferation in vitro and in vivo. **a** Verification of the efficiencies of CD9 and CD81 overexpression in Huh7 and Hep3B cells via western blotting. **b** CCK-8 assays were performed to evaluate cell proliferation rates of HCC cells described in (**a**), the absorbance at 450 nm was measured at the indicated time points. **c** Colony formation assays were employed using Huh7 and Hep3B cells transduced with LV-Vec, LV-CD9 or LV-CD81 lentiviruses, respectively. **d** EdU-based assays were performed to determine the effects of CD9 and CD81 on HCC cell proliferation. Representative images were shown and the percentage of EdU-positive cells were calculated and exhibited in the column graph. Scale bar = 100 μm. **e** Huh7 cells stably infected with LV-Vec, LV-CD9 or LV-CD81 lentiviruses were injected subcutaneously into the armpit regions of nude mice, respectively (*n* = 6) . After 4 weeks, the tumors were removed and weighted. The pictures of gross tumors and tumor weights were shown. *P < 0.05, **P < 0.01.

To explore the role of altered CD9/CD81 expression in HCC progression, Huh7 and Hep3B cells were successfully transfected with CD9 or CD81-overexpression plasmids (Fig. 3a), and cell proliferation assays were conducted. As the growth curves shown in Fig. 3b, overexpression of CD9 and CD81 significantly suppressed proliferation rates of HCC cells. Besides, HCC cell lines with stable overexpression of CD9 and CD81 were established and colony formation assays were employed. The number of colonies formed from cells with stable overexpression of CD9 and CD81 were obviously fewer than their control groups (Fig. 3c). As the same time, the results of EdU assays showed that overexpression of CD9 and CD81 remarkably decreased the percentage of EdU-positive cells, indicating that CD9 and CD81 suppressed HCC cell proliferation (Fig. 3d). Moreover, to confirm these findings in vivo, CD9- or CD81-overexpressed Huh7 cells were used to establish a subcutaneous xenotransplanted tumor model in nude mice. The results showed that both of CD9 and CD81 overexpression significantly inhibited tumor growth (Fig. 3e), which were in line with the results in vitro. Collectively, these observations implied that CD9 and CD81 may function as tumor suppressors in HCC.

### Knockdown of CD9 and CD81 results in enhancement of HCC cell growth

**Fig. 4:**
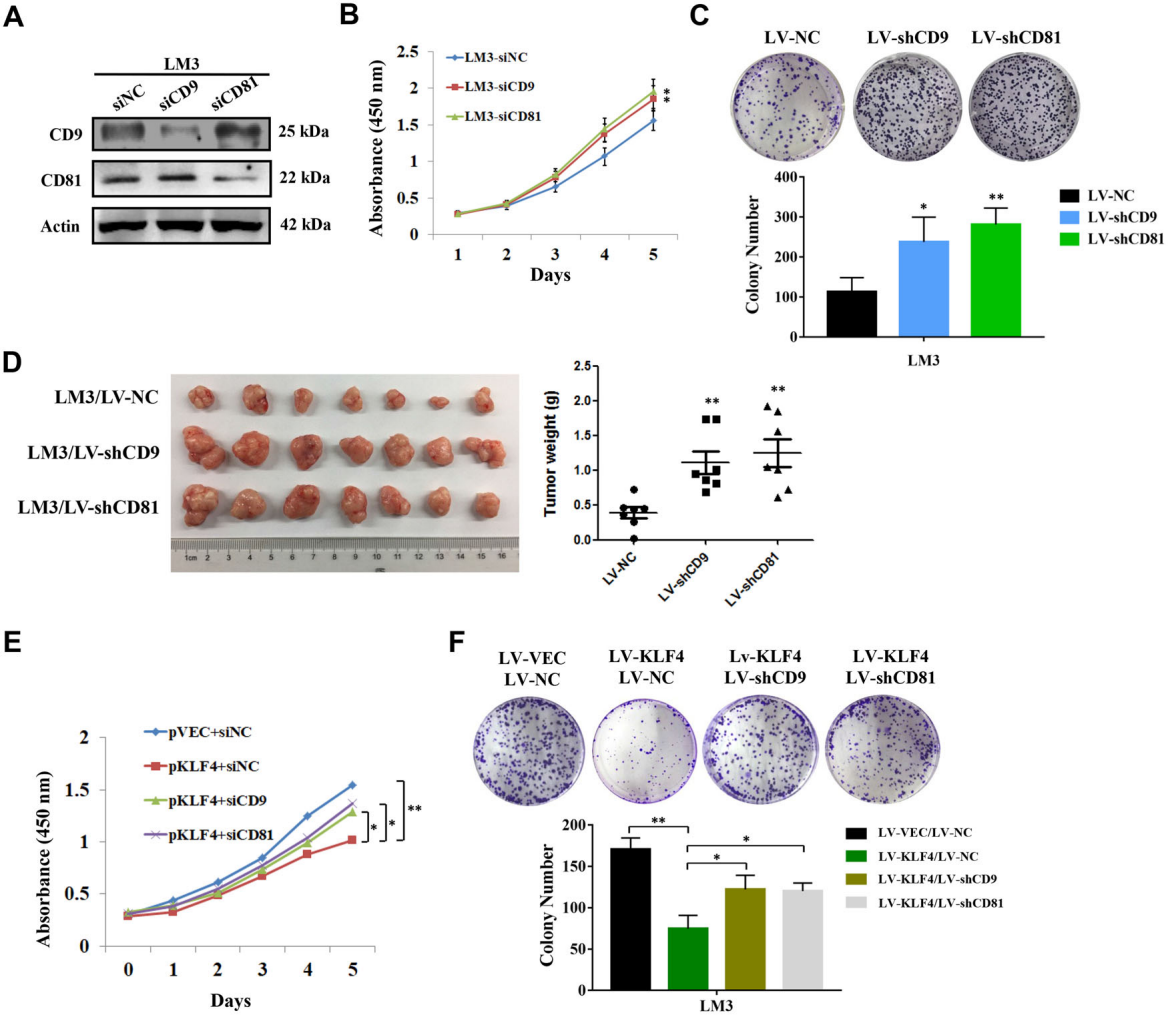
Silenced CD9 and CD81 expression contributes to cell growth and tumorigenicity of HCC cells. **a** Verification the efficiencies of the siRNAs against CD9 and CD81 by western blotting in HCC-LM3 cells. **b** Cell growth curves indicating higher proliferative ability of HCC-LM3 cells transfected with siCD9 or siCD81 comparing with the control group. **c** Colony formation assays were performed after HCC-LM3 cells were stably infected with LV-NC, LV-shCD9, LV-shCD81 lentiviruses. Representative images of colonies were shown and colony number of each group was calculated. **d** HCC**-**LM3 cells described in **c** were implanted subcutaneously into the armpit regions of nude mice, respectively (*n* = 7). Tumors were removed and weighted after 4 weeks. Pictures of gross tumors and tumor weights of each group were presented. **e** siNC, siCD9 or siCD81 were transfected into KLF4-overexpressed HCC-LM3 cells and CCK-8 assays were performed. **f** Colony formation assays were performed to determine whether CD9 or CD81 mediated KLF4-induced HCC cell proliferation suppression. *P < 0.05, **P < 0.01.

To further confirm the effects of CD9/CD81 expression on HCC cell proliferation, HCC-LM3 cells were transiently transfected with siRNAs against CD9 or CD81 respectively and the knockdown efficiencies were validated by western blotting (Fig. 4a). As shown in Fig. 4b, cells with reduced CD9/CD81 expression exhibited higher proliferation rates in CCK-8 assays. In the meantime, no significant additive effect of double knockdown of CD9 and CD81 was observed (Supplementary Fig. 2). Furthermore, stably silenced CD9 or CD81 expression led to more colonies formation in HCC-LM3 cells (Fig. 4c). The results of subcutaneously transplanted tumor model indicated that CD9 or CD81 knockdown significantly enhanced tumorigenicity of HCC in vivo (Fig. 4d). To detect the functional relevance between KLF4 and CD9/CD81, we overexpressed KLF4 in CD9 or CD81 knockdown cells. The final results from CCK-8 assays and colony formation assays indicated that knockdown of CD9 or CD81 notably attenuated the inhibitory effects induced by KLF4 overexpression (Fig. 4e and f), hinting that CD9 and CD81 may functionally act as downstream effectors of KLF4 during HCC cell proliferation.

### CD9 and CD81 regulate JNK-Cyclin D1/Bcl2 signaling in HCC cells

**Fig. 5:**
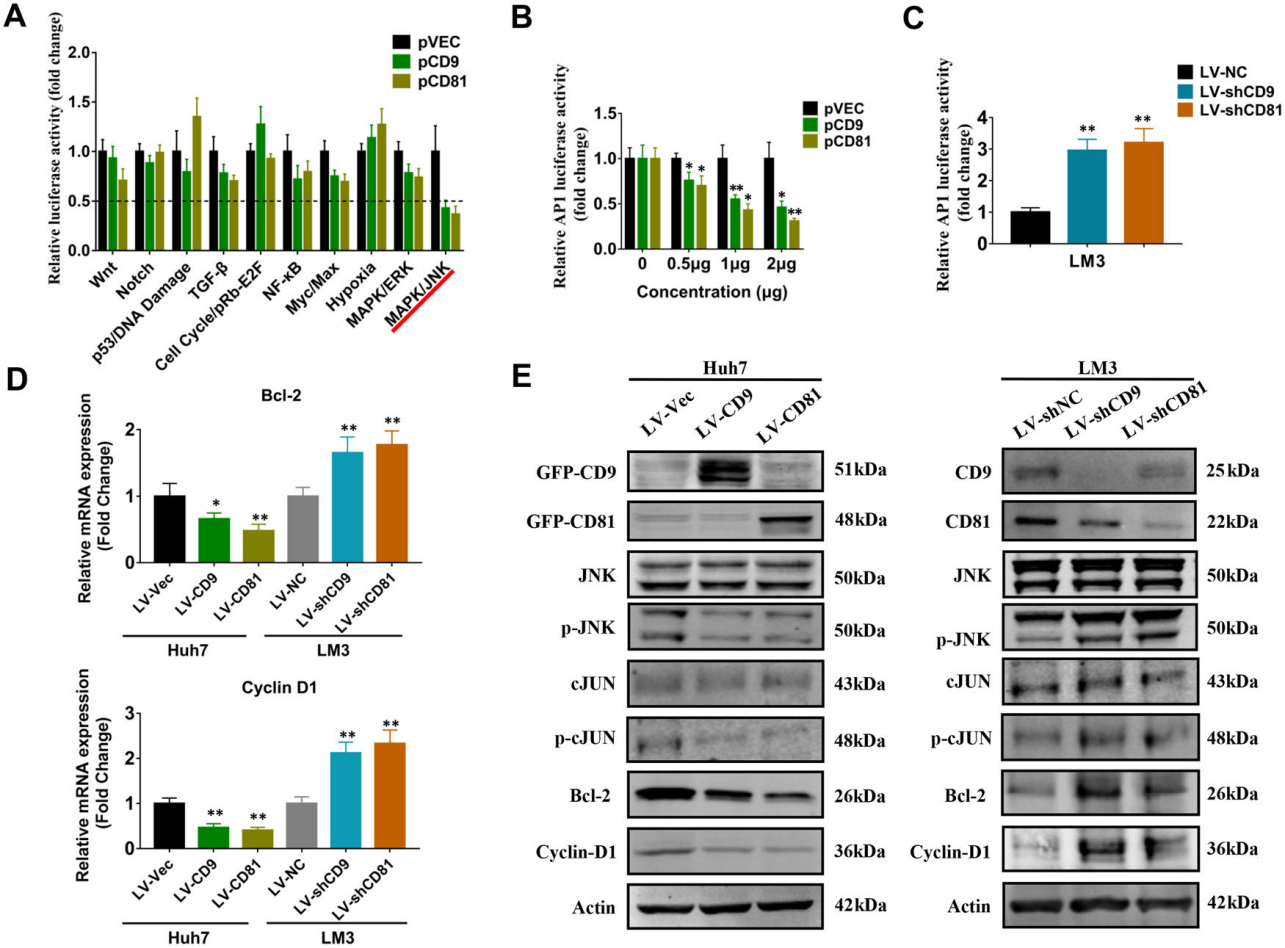
Effects of CD9 and CD81 expression on the activity of JNK signaling pathway. **a** Cignal Finder Reporter Array was conducted in Huh7 cells transfected with CD9 or CD81 constructs. **b**, **c** Dual Luciferase reporter assays were performed using Huh7 (**b**) and HCC-LM3 (**c**) cells to determine whether CD9 or CD81 could affect the promoter activity of AP-1. **d** qPCR analyses were employed to determine whether CD9 or CD81 regulated Cyclin D1 and Bcl-2 mRNA expression level in Huh7 and HCC-LM3 cells. **e** Western blots were performed to verify the effects of altered CD9 or CD81 expression on the expression of specific proteins related with JNK signaling pathway. *P < 0.05, **P < 0.01.

To investigate the regulatory mechanisms of CD9 and CD81 in HCC cell proliferation, Cignal Finder Cancer 10-Pathway Reporter Array was performed. Of the 10 pathways tested, both CD9 and CD81 overexpression in Huh7 cells significantly impinged on TGF-β, NF-κB, Myc/Max and MAPK/JNK signaling pathways. Using a more stringent criteria (fold change < 0.5, *P* < 0.01 for both of CD9 and CD81 overexpression), MAPK/JNK pathway was identified to be the most heavily affected pathway by CD9 and CD81 overexpression (Fig. 5a). To validate the results, luciferase reporter assay was performed to determine whether CD9/CD81 alteration could influence the transcriptional activity of Activating Protein 1 (AP-1), a downstream transcription factor of JNK pathway mainly comprised by c-JUN and c-Fos family numbers^34^. Consistently, CD9/CD81 overexpression resulted in inhibition of AP-1 response element activity in a dosage-dependent manner (Fig. 5b). Conversely, knockdown of CD9/CD81 remarkably enhanced AP-1 response element activity (Fig. 5c). These results suggested that CD9/CD81 could regulate JNK/AP-1 signaling pathway in HCC cells. Emerging evidences have shown that JNK pathway promotes cancer cell proliferation and inhibits cell apoptosis via regulating its downstream genes such as Cyclin D1^35^ and Bcl-2^36^. Accordingly, we performed qPCR analyses to examine the effects of CD9/CD81 alteration on the expression of Bcl-2 and Cyclin D1. As shown in Fig. 5d, overexpression of CD9 and CD81 significantly decreased the expression level of Cyclin D1 and Bcl-2, while silencing of their expression led to opposite results. In addition, western blot analysis was performed to investigate the expression of specific proteins related with JNK signaling pathway. The results demonstrated that CD9/CD81 overexpression strongly inhibited the phosphorylation of JNK and c-JUN, while total JNK and c-JUN expression exhibited no difference compared with the control group, and expression of Bcl-2 and Cyclin D1 were suppressed as well. On the contrary, silencing CD9 and CD81 expression led to opposite consequences in contrast to overexpression of CD9 and CD81 (Fig. 5e). Therefore, our results indicated that reduced expression of CD9 and CD81 in HCC may activate JNK signaling pathway, which promotes cancer cell proliferation via regulating the downstream genes such as Cyclin D1 and Bcl-2.

### Effects of KLF4-CD9/CD81 signaling on exosome secretion of HCC cells

**Fig. 6:**
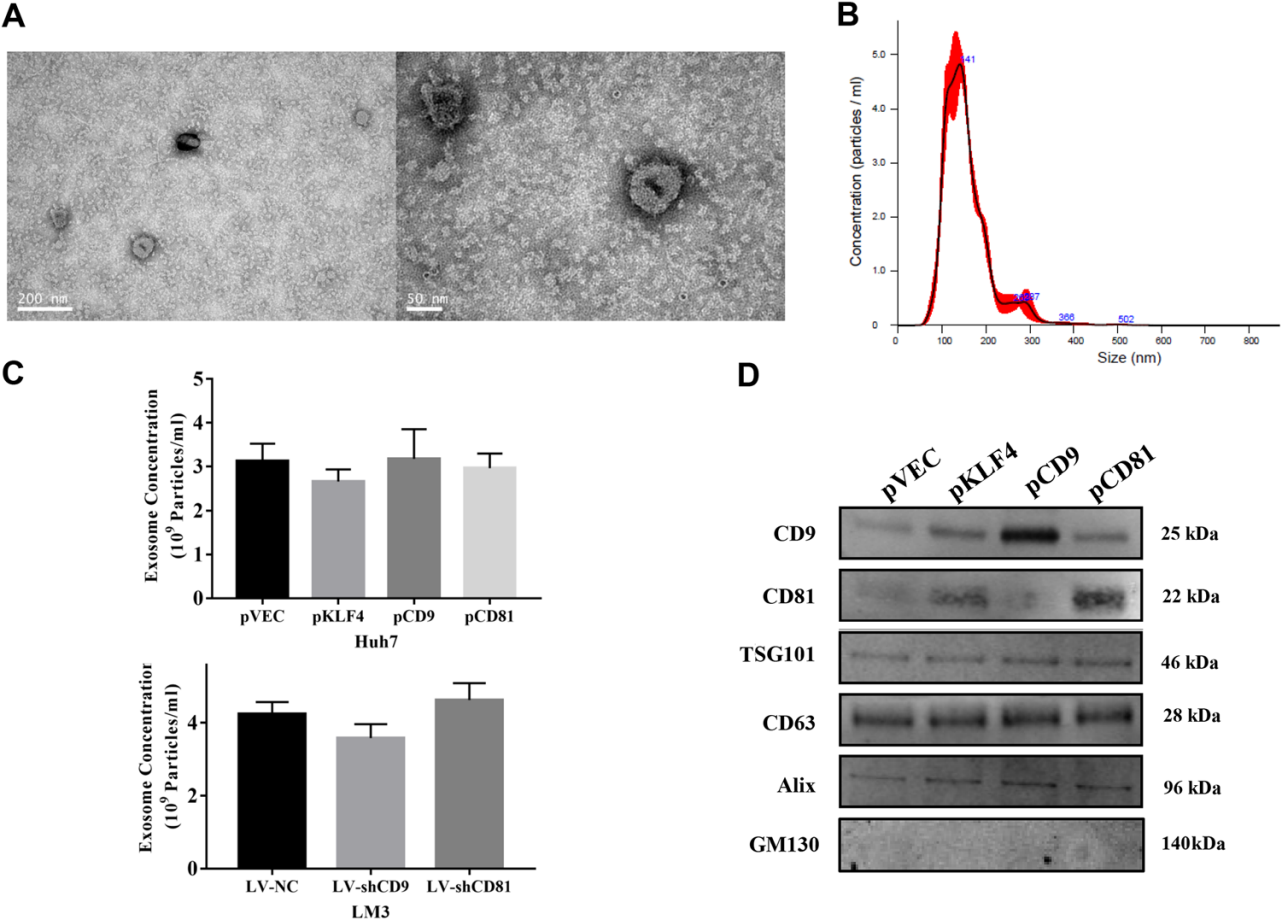
KLF4, CD9 or CD81 has no significant effect on HCC-derived exosome abundance. **a**, **b** Exosomes isolated from the cell culture medium of Huh7 or HCC-LM3 cells were analyzed by TEM and NTA. Representative pictures of TEM (**a**) and NTA (**b**) were shown. **c** Results of NTA indicating that altered expression of KLF4, CD9 or CD81 didn’t affect the abundance of HCC-secreted exosomes. **d** Western blots were performed using the exosomes isolated from Huh7 cells transfected with pVEC, pKL4, pCD9 or pCD81. GM130 was used as a negative control. TEM, transmission electron microscopy. NTA, Nanoparticle tracking analysis.

Given that CD9 and CD81 are identified as exosomal biomarkers, we further analyzed whether the regulation of CD9/CD81 by KLF4 influences exosome secretion of HCC cells. To determine the impacts of altered KLF4, CD9, and CD81 expression on exosome secretion, exosomes were isolated from culture medium of Huh7 and HCC-LM3 cells via ultracentrifugation method, after which the exosomes were visualized using transmission electron microscopy. As the pictures shown in Fig. 6a, the EVs with the expected size range of exosomes (50–150 nm) and a double membrane confirmed the presence of exosomes. Similarly, results of NTA analysis indicated that the size of purified exosomes were approximately 150 nm (Fig. 6b). However, our results showed that altered expression of KLF4, CD9 or CD81 had no obvious influence on the concentration of exosomes secreted from HCC cells (Fig. 6c). To validate the results of NTA analysis, we performed western blot analysis using the exosomes isolated from culture medium of HCC cells, and CD9, CD63, CD81, Alix, and TSG101 protein expression were examined. We observed that the abundances of CD9 and CD81 in the exosomes were significantly upregulated after overexpression of KLF4 or themselves in HCC cells, however, the upregulation of KLF4, CD9 or CD81 had no influence on CD63, Alix and TSG101 expression (Fig. 6d), implying that instead of affecting the total number of secreted exosomes, KLF4-CD9/CD81 signaling might change the subtypes of exosomes, which finally influenced the functions of HCC-derived exosomes.

## Discussion

The exosomal surface proteins CD9 and CD81 belong to the tetraspanin family, which can form a tetraspanin web to interact with other cell surface molecules. More and more evidences have suggested their key roles in many physiological and pathological processes^14,37,38^. Some previous studies have shown that CD9 and CD81 are involved in cancer. For example, CD9 inhibits the proliferation and tumorigenicity of human colon carcinoma cells^39^, and reduced CD9 expression is associated with poor prognosis of different cancer types^40–42^. CD81 acts as a tumor suppressor gene in gastric cancer^43^, and low expression of CD81 in HCC cell lines has more metastatic potential than those with higher CD81 expression^44^. A more recent study reveals that CD81 suppression promotes bladder cancer cell invasion through increased matrix metalloproteinase (MMP) expression^45^. Other studies have also indicated the oncogenic roles of CD9 and CD81 in several cancer types^46,47^. However, the exact function of CD9 and CD81 in HCC remains to be elucidated. In our study, we provide evidence that CD9 and CD81 inhibit HCC cell proliferation in vitro and in vivo, although we did not observe any effects of CD9 and CD81 overexpression or knockdown on cell migration.

Several researches have revealed altered CD9 or CD81 expression in other types of cancer, but to date, little is known about the mechanisms which cause the changes of CD9 and CD81 expression during cancer initiation and metastasis. Our work demonstrated that both CD9 and CD81 are directly transcriptional targets of KLF4, which has been recognized and addressed as a tumor suppressor in various cancers. IHC staining and qPCR results demonstrated that CD9 and CD81 are frequently decreased in HCC specimens and correlated with KLF4 expression, supporting the regulation of CD9 and CD81 by KLF4. Researchers have reported that CD9 and CD81 could interact with specific genes such as MMPs and epidermal growth factor receptor (EGFR), and reduced expression of CD9 was found to activate JNK signaling pathway in kerationcytes. Coincidentally, our results of Cignal Finder Cancer 10-Pathway Reporter Arrays and western blots demonstrated that both CD9 and CD81 overexpression remarkably represses the phosphorylation of JNK and as well as c-JUN, the prototypical downstream target of JNK, suggesting that CD9 and CD81 contribute to the inhibition of JNK signaling pathway.

**Fig. 7:**
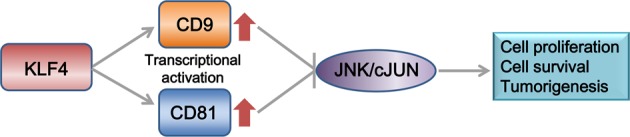
A schematic diagram of regulatory relationship among KFL4. CD9/CD81 and JNK signaling.

The JNK pathway regulates key cellular activities involved in cancer, persistent activation of JNKs (JNK1, JNK2, and JNK3) can influence tumor initiation and development by either transcription-dependent or transcription-independent mechanisms related with cell proliferation, apoptosis, autophagy and survival^48^. Cyclin D1 and Bcl-2 have been recognized as downstream effectors of JNK signaling^35,36^. Consistently, our results of qPCR and western blot analyses also found that reduced expression of CD9 and CD81 activates the JNK pathway, as well as enhances the expression of cyclin D1 and Bcl-2, which in turn promoted HCC cell proliferation and anti-apoptosis. The possible regulatory axis among KLF4, CD9/81, and JNK signaling is depicted in Fig. 7. But how CD9 and CD81 regulate the JNK pathway, whether they function as a complex and specific functions of their downstream target genes, remain unclear currently and requires further experiments.

Over the past decades, exosomes are increasingly recognized as critical carriers of information related with tumor microenvironment (TME) regulation and as pivotal roles in tumorigenic activities such as tumor survival, proliferation, angiogenesis in various human malignancies, including HCC^5,7,49^. For instance, Takayuki et al. found that exosomes packed with miRNAs derived from HCC cells regulate tumor progression by targeting TGF-β activated kinase 1 (TAK1)^50^. Both of CD9 and CD81 are expressed on the surface of exosomes and usually used to represent the populations of exosomes, suggesting that KLF4-CD9/CD81 signaling is involved in exosome biogenesis or secretion of HCC. By NTA and western blot analyses, we reported that KLF4 overexpression significantly increased the abundance of CD9 and CD81 in exosomes secreted from HCC cells, even though it didn’t alter the abundance of secreted exosomes. Recently, scientists have proposed some suggestions to classify EVs into several subtypes using different biomarkers, and exosomes enriched in tetrapsanins CD63, CD9, CD81 are defined as a small EV subtype, although this definition still requires further validation^51^. Hence, Combined with our data, it is reasonable to hypothesize that KLF4 might regulate the subtypes of HCC-secreted exosomes, which influences their functions in cell-to-cell communication, and finally participates in HCC development. However, this hypothesis needs to be validated in future studies.

In summary, our findings have identified CD9 and CD81 as potential tumor suppressor genes in HCC, which may mediate KLF4-induced suppression of tumor cell growth via JNK signaling for the first time. On the other hand, KLF4-triggered upregulation of CD9 and CD81 appears no effect on exosomal abundance secreted from HCC cells. These results provide new insights into exosomal surface markers in tumor development and regulatory mechanism underlying HCC development.

## Materials and methods

### Human HCC tissue specimens

Thirty-four-paired tumor and adjacent normal tissues were collected from patients with previously untreated HCC who accepted curative surgery at Shanghai East Hospital in Shanghai, China, from January 2013 to April 2014. The tissue specimens were snap-frozen and stored in liquid nitrogen before RNA extraction. Informed consent was obtained from each patient prior to sample collection. This study was approved by the Ethics Committee of Shanghai East Hospital.

### Tissue microarray and immunohistochemical analysis

A human HCC tissue microarray (#HLivH150CS03) purchased from Shanghai Outdo Biotech CO., LTD., China was prepared for immunohistochemical analysis. 75 patients with detailed clinical information were enrolled in this tissue microarray. Among these patients, 62 are male, 13 are female, and the mean age is 56.5 years old (range from 40 to 73 years old). To detect the expression of KLF4, CD9 and CD81 protein in HCC tumor and adjacent normal tissues, standard immunohistochemical procedures were performed using specific antibodies against KLF4 (dilution, 1:500; #11880-1-AP; Proteintech), CD9 (dilution, 1:500; #20597-1-AP; Proteintech), and CD81 (dilution, 1:500; #14387-1-AP; Proteintech). The staining results were evaluated by two pathologists blinded to the clinical information independently.

### Cell culture

Human HCC cell lines Huh7, Hep3B, HCC-LM3, Sk-Hep-1, and a fetal liver-derived cell line L02 were purchased from Shanghai Cell Bank of the Chinese Academy of Sciences Shanghai, China. All of cells were maintained in Dulbecco’s modified Eagle’s medium (DMEM, Corning, Inc., Corning, NY, USA) supplemented with 10% fetal bovine serum (FBS) and 1% penicillin/streptomycin (M&C Gene Technology Ltd.). Cells were incubated in a thermostatic incubator at 37 °C with 5% CO_2_.

### Cell transfection

The full length cDNA of CD9 (GeneBank accession no. NM_001769.4) or CD81 (GeneBank accession no. NM_004356.3) was respectively cloned into the pcDNA3.1 expression vector, and the sequences were confirmed by DNA sequencing. The FLAG-tagged KLF4-expression plasmid was a gift from Dr. Keping Xie (MD Anderson Cancer Center). The siRNAs against KLF4, CD9 and CD81 were synthesized by GenePharma Co., Ltd., Shanghai, China. HCC cells were transfected with plasmids or siRNAs using Lipofectamine 3000 (Invitrogen, USA) according to the protocol of manufactures. The sense sequences of siRNAs are as follows: siKLF4: 5′-UUGTGGAUAUCAGGGUAUAAA-3′, siCD9: 5′-GUGGAACAGUUUAUCUCAUdTdT-3′, siCD81: 5′- UGAUGUUCGUUGGCUUCCUUU-3′. For stable knockdown of the three genes, LV-shKLF4, LV-shCD9, LV-shCD81 lentiviral particles were purchased from GenePharma, Shanghai matching above siRNA sequences. For CD9, CD81 or KLF4 overexpression, lentiviral particles containing CD9-GFP or CD81-GFP were purchased from Shanghai Sunbio Medical Biotechnology, and KLF4 overexpression lentivirus was purchased from Shanghai Obio Technology Corporation. HCC cells were infected with the indicated lentivirus with the assistance of polybrene (4 μg /mL). Stable cell lines were selected using puromycin (2.5 μg/mL).

### Western blot analysis

Cells were lysed in RIPA buffer supplemented with protease inhibitor cocktail (Sigma Aldrich; Merck KGaA, Darmstadt, Germany). The protein extracts were separated by SDS-PAGE and then transferred onto a polyvinylidene difluoride (PVDF) membrane. The membrane was blocked with 5% non-fat milk in 0.1% Tween-20 in PBS (PBST) for 1 h at room temperature, and after incubation with appropriate primary antibodies overnight at 4 °C, the membrane was incubated with the corresponding secondary antibodies for 1 h, and a Li-Cor Odyssey protein imaging system (LI-COR Biosciences, Lincoln, NE, USA) was used to detect the immunoreactive signal and analyze the protein bands. Antibodies used in the present study are as follows: Anti-CD9 (1:500, Proteintech, #20597-1-AP), anti-CD81 (1:500, Proteintech, #14387-1-AP), anti-CD63 (1:200, Santa Cruz Biotechnology, #sc-365604), anti-FLAG (1:2000, Sigma-Aldrich, #F1804), anti-Alix (1:500, Proteintech, #12422-1-AP), anti-TSG101 (1:500, Proteintech, #28283-1-AP), anti-GM130 (1:800, Cell Signaling Technology, #12480), anti-β-actin (1:1000, Santa Cruz Biotechnology, #sc-81178), anti-GFP (1:500, Proteintech, #50430-2-AP), anti-JNK (1:1000, Cell Signaling Technology, #9252), anti-p-JNK (1:1000, Cell Signaling Technology, #4668), anti-cJUN (1:500, Cell Signaling Technology, #9165), anti-p-cJUN (1:500, Cell Signaling Technology, #2361), anti-Bcl-2 (1:500, Cell Signaling Technology,#4223), anti Cyclin-D1 (1:500, Cell Signaling Technology, #2978).

### Total RNA isolation and quantitative real-time polymerase chain reaction (qPCR) analysis

Total RNA from the HCC cell lines was isolated with Trizol reagent (Sigma Aldrich, MO, USA) according to the manufacturer’s protocol. A Fisher Scientific NanoDrop™ 2000 spectrophotometer (NanoDrop; Thermo Fisher Scientific, Inc., Wilmington, DE, USA) was used to investigate the quality and concentration of RNA, and 1 μg total RNA was used to synthesize cDNA using the PrimeScript™ RT reagent kit (Takara Bio, Inc., Otsu, Japan) prior to qPCR analysis. SYBR green reagent (TaKaRa, Japan) was used to perform qPCR analysis on an ABI QuantStudio^TM^ 6 Flex system (Thermo Fisher Scientific, Inc.). *β*-actin was used as an internal control, and relative mRNA expression level was quantified using the 2^−ΔΔCt^ method. The qPCR primers used in this study were included in Supplementary Table 2.

### Colony formation assay

HCC cells infected with lentiviruses were trypsinized to prepare monoplast suspension, then 1 × 10^3^ of the cells were seeded into a 6-well plate and incubated under normal conditions for 2 weeks. The colonies were fixed using 4% paraformaldehyde for 20 min at room temperature and stained with crystal violet for 15 min at room temperature. The colonies were photographed, and Image J software was used to count the number of colonies. Each experiment was performed in triplicate and repeated at least three times.

### Cell proliferation assay

Cell Counting Kit-8 (CCK8, Dojindo, Kumamoto, Japan) was used to examine proliferation ability of HCC cells. Briefly, cells were seeded into a 96-well plate at a density of 3 × 10^3^ cells per well and cultured under normal conditions. At the scheduled time points (0, 24, 48, 72, 96 h), 10 μl CCK8 reagents were added into each well and incubated for 1 h at 37 °C. An automated microplate reader (SpectraMax M5, Molecular Devices LLC, CA, USA) was used to measure the absorbance at the wavelength of 450 nm according to the manufacture’s specifications. Each experiment was performed in triplicate and repeated at least three times.

### 5′-ethynl-2′-deoxyuridine (EdU) cell proliferation assay

EdU Cell Proliferation Assay Kit (Ribobio, Guangzhou, China) was performed to investigate HCC cell proliferation ability. Briefly, HCC cells stably infected with lentiviruses were seeded into a 12-well plate at a density of 5 × 10^5^ cells per well and cultured under standard conditions for 24 h, after which the cells were stained with EdU for 2 h at room temperature and then Apollo 567 for 30 min shielded from light, and the cell nuclei were stained with 1 μg/mL DAPI for 10 min. The proportion of EdU-positive cells was determined under a fluorescence microscope (Leica DMI3000B, Leica Microsystems, Inc.). The experiment was performed in triplicate and repeated at least three times.

### Measurement of promoter reporter activity by dual luciferase assay

Wild or mutant CD9/CD81 promoter was cloned into pGL3 basic luciferase reporter vector, respectively. The products were verified by DNA sequencing. AP-1-Luc reporter plasmid (AP-1 response element construct) was purchased from Genomeditech, Shanghai, China. To perform dual-luciferase assay, HCC cell lines in exponential growth phase were seeded into a 24-well plate and transfected with the indicated luciferase reporters (or KLF4 overexpression plasmid and siKLF4). Twenty-four hours after transfection, the luciferase activity was measured by quantifying both firefly and Renilla luciferase activity using the Dual-Glo® Luciferase Assay System (Promega, WI, USA) following the specifications.

### Cignal finder cancer 10-pathway reporter array

To explore the pathways regulated by CD9/CD81, Cignal Finder 10-Pathway Reporter Array (Qiagen, Dusseldorf, Germany) was used according to the instructions. Briefly, 50 μl cell suspension (8 × 10^4^ cells maintained in Opti-MEM® with 10% FBS) was seeded to each well after preparation of complex formation and incubated for 16 h. After incubation, previous medium was replaced with complete growth medium. Finally, dual luciferase assay was carried out using the Dual-Glo® Luciferase Assay System (Promega, WI, USA), and the results were analyzed on a Glomax-multi Luminometer (Promega, WI, USA).

### Animal experiments

To establish subcutaneous xenograft model, 4-week old, male pathogen-free athymic nude mice (BALB/c) were purchased from Sippr-BK Lab Animal Co., Ltd., Shanghai, China. Equal amounts of human HCC cell lines (HCC-LM3: 2 × 10^6^ cells per tumor, *n* = 7; Huh7: 3 × 10^6^ cells per tumor, *n* = 6) were injected subcutaneously into the left or right armpit regions of nude mice. The tumor-bearing mice were bred under normal feeding conditions for 4 weeks and then euthanized to remove the tumors. The tumors were weighted and photographed. All animal handling and experimental protocol in this study were also approved by the Ethics Committee of Shanghai East Hospital.

### Chromatin immunoprecipitation (ChIP) assay

Huh7 and L02 cells (2 × 10^6^) were prepared for ChIP assay using a ChIP assay kit (Millipore, CA, USA) as per the manufacturer’s instructions. The precipitated DNA samples obtained from the steps above were subjected to PCR in order to amplify fractions of the promoters of CD9 and CD81. Afterwards, the PCR products were resolved electrophoretically with a 2% agarose gel and the results were visualized using the ChemiDoc MP system (Bio-Rad Laboratories, Hercules, CA, USA). The primers used for ChIP assays were listed in Table S1.

### Exosome isolation

Exosomes from HCC cell culture medium were isolated by ultracentrifugation method. Briefly, cell medium was collected into a 50 ml centrifuge tube and centrifuged at 1500 × *g* for 5 min and then 10,000 × *g* for 30 min. The debris was removed and supernatant was immediately filtered using a filter unit with a 0.22 μm membrane to remove intact cells and cell debris, then ultracentrifugation was performed at 150,000 × *g* for 16 h, and the pellet was resuspended in 1 mL of PBS and ultracentrifuged again at 150,000 × *g* for 2 h at 4 °C. The pallet was purified by density gradient ultracentrifugation and either used immediately or stored at −80 °C. An optimal L-100XP ultracentrifuge (Beckman Coulter, Brea, USA) was used to perform the ultracentrifugation steps above.

### Nanoparticle tracking analysis

To directly analyze the number and size of the exosomes extracted from cell culture medium, the NanoSight NS 300 system (NanoSight Technology, Malvern, UK) was used. The samples were diluted 150 times with sterile PBS prior to each analysis. Particle diameter was calculated according to velocity of Brownian motion.

### Transmission electron microscopy (TEM)

Exosome samples re-suspended in PBS were dropped onto a formvar-carbon coated copper grid and left to dry for 30 min at room temperature. The samples were fixed by 2% paraformaldehyde for 10 min, followed by five washes in purified water for 3 min. After fixation, the grids were stained with 2% uranyl acetate for contrast enhancement. The grids were visualized using a transmission electron microscope (JEM 1400, Jeol, Peabody, MA, USA) and the images were captured.

### Statistical analysis

Quantitative data were represented as mean ± standard error of the mean (SEM) of at least three independent repeated experiments. GraphPad Prism 7.0 software was used to perform statistical analysis. Categorical data was analyzed using *χ*^2^ test, the significance of the data from independent experiments were determined using the two-tailed Student *t*-test or one-way analysis of variance (ANOVA). *P* values <0.05 were considered statistically significant.

## Supplementary information

Supplementary figure and table legends

Supplementary Table 1

Supplementary Table 2

Supplementary Figure 1

Supplementary Figure 2
