# Supplementary material for: KLF4-mediated upregulation of CD9 and CD81 suppresses hepatocellular carcinoma development via JNK signaling
Source: Cell Death Dis. 2020 Apr 29;11(4):299. doi: 10.1038/s41419-020-2479-z (PMC7190708; doi:10.1038/s41419-020-2479-z)
Supplement: Supplementary file 1 — Supplementary figure and table legends [file 41419_2020_2479_MOESM1_ESM.docx]

**Supplementary Figure 1:** **The expression of KLF4 in HCC samples.** **a**. Representative images of KFL4 staining in HCC tumor tissues and adjacent normal tissues. Magnification: 100× and 400×. Scale bar = 50μm. **b**. Column graphs indicate the case number of different KLF4 staining intensity.

**Supplementary Figure 2: Altered expression of CD9 and CD81 has an impact on HCC cell growth.** The effects of silenced CD9 or CD81 on HCC-LM3 cell growth. Cell growth was measured by CCK-8 assays. **P* < 0.05, ***P* < 0.01.

**Supplementary Table 1:** The correlation of KLF4 expression with clinicopathological parameters of HCC patients.

**Supplementary Table 2**: Primers used for qPCR and ChIP assay.
