# Supplementary material for: KLF4-mediated upregulation of CD9 and CD81 suppresses hepatocellular carcinoma development via JNK signaling
Source: Cell Death Dis. 2020 Apr 29;11(4):299. doi: 10.1038/s41419-020-2479-z (PMC7190708; doi:10.1038/s41419-020-2479-z)
Supplement: Supplementary file 2 — Supplementary Table 1 [file 41419_2020_2479_MOESM2_ESM.docx]

| Supplementary Table 1：The correlation of KLF4 expression with clinicopathological parameters of HCC patients | | | | |
| --- | --- | --- | --- | --- |
|  | Number of  cases | Negative,  weak | Moderate,  strong | *P* value |
| Age (years) |  |  |  |  |
| > 60 | 18 | 15 | 3 | >0.05 |
| ≤60 | 57 | 47 | 10 |  |
| Gender |  |  |  |  |
| Male | 62 | 52 | 10 | >0.05 |
| Female | 13 | 10 | 3 |  |
| Grading |  |  |  |  |
| I,II | 64 | 53 | 11 | >0.05 |
| III,IV | 11 | 9 | 2 |  |
| Metastasis |  |  |  |  |
| M0 | 71 | 60 | 11 | >0.05 |
| M1 | 4 | 2 | 2 |  |
| TNM Stage |  |  |  |  |
| I ,II | 45 | 41 | 4 | 0.018 |
| III, IV | 30 | 21 | 9 |  |
