# Supplementary material for: KLF4-mediated upregulation of CD9 and CD81 suppresses hepatocellular carcinoma development via JNK signaling
Source: Cell Death Dis. 2020 Apr 29;11(4):299. doi: 10.1038/s41419-020-2479-z (PMC7190708; doi:10.1038/s41419-020-2479-z)
Supplement: Supplementary file 3 — Supplementary Table 2 [file 41419_2020_2479_MOESM3_ESM.docx]

**Supplementary Table 2**: **Primers used for qPCR and ChIP assay.**

**Sequences of primers for qPCR analysis**

| **Gene** **Sequence** |
| --- |
| KLF4 5’-AGAGGAGCCCAAGCCAAAG-3’  5’-CGTCCCAGTCACAGTGGTAAG-3’ |
| CD9 5’-GGACGTACTCGAAACCTTCACC-3’  5’-GCGGATAGCACAGCACAAGA-3’ |
| CD81 5’-CTGACTGCTTTGACCACCTCA-3’  5’-AATGCCGATGAGGTACAGCTT-3’ |
| Cyclin D1 5’-GGATGCTGGAGGTCTGCGA-3’  5’-TAGAGGCCACGAACATGCAAGT-3’ |
| Bcl-2 5’-ATAACGGAGGCTGGGATGCCTT-3’  5’-GCAGGCATGTTGACTTCACTTG-3’ |
| β-actin 5’CCTGGCACCCAGCACAATG-3’  5’GGGCCGGACTCGTCATACT-3’ |

**Sequences of primers for ChIP assays**

| **Gene** **Sequence** |
| --- |
| CD9#1 5’-cctctccttagcctcttag-3’  5’-gtagacttgtctcattggttc-3’ |
| CD81#2 5’-aaccttcgtcagtgtgtca-3’  5’-tggctcttccggaaggtgt-3’ |
| CD81#3 5’-agacgctgcatgcctgt-3’  5’-cccaggtgcaccgtgct-3’ |
