# Supplementary figures and images for: KLF4-mediated upregulation of CD9 and CD81 suppresses hepatocellular carcinoma development via JNK signaling
Source: Cell Death Dis. 2020 Apr 29;11(4):299. doi: 10.1038/s41419-020-2479-z (PMC7190708; doi:10.1038/s41419-020-2479-z)

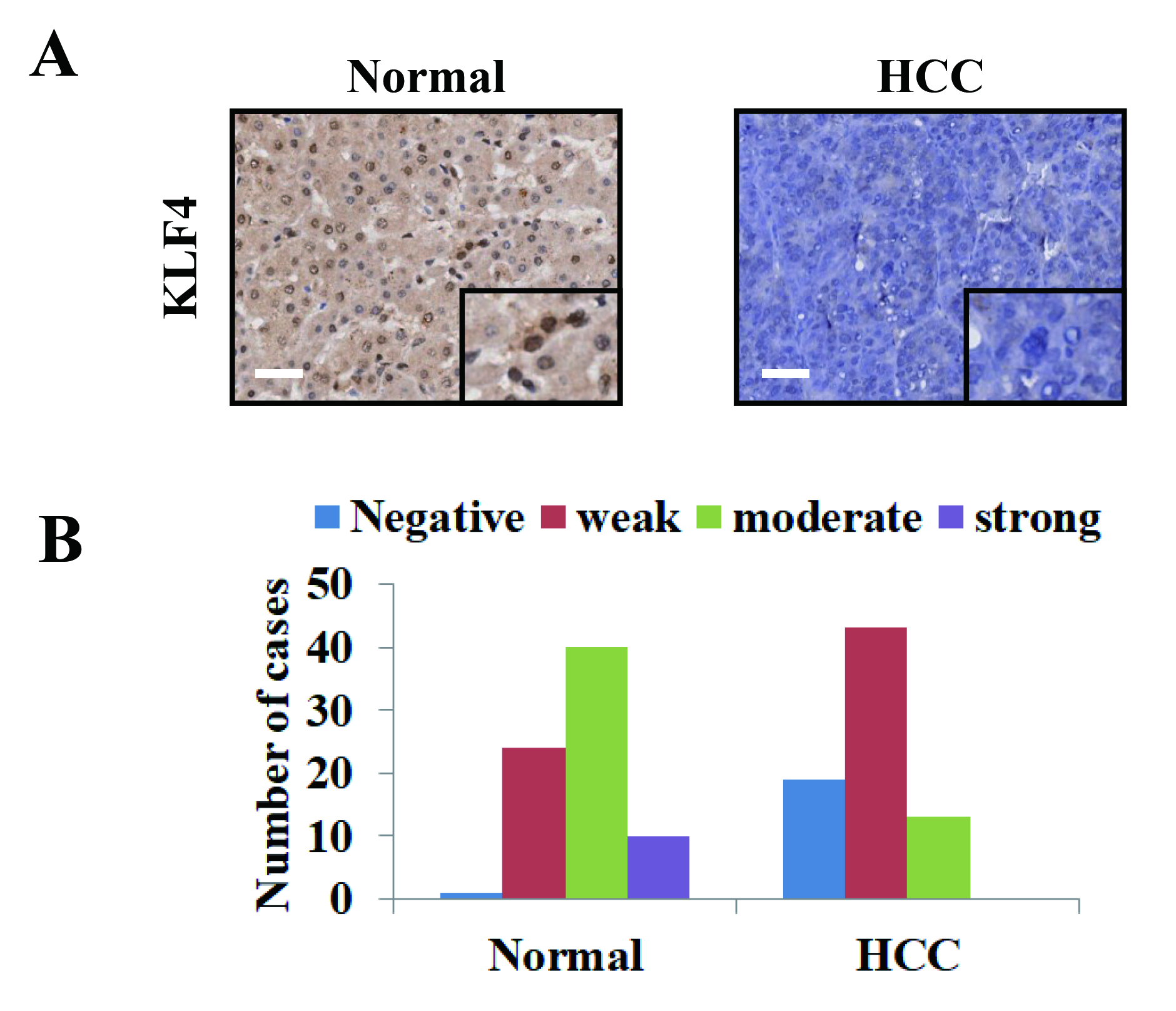

Supplement: Supplementary file 4 — Supplementary Figure 1 [file 41419_2020_2479_MOESM4_ESM.tif]

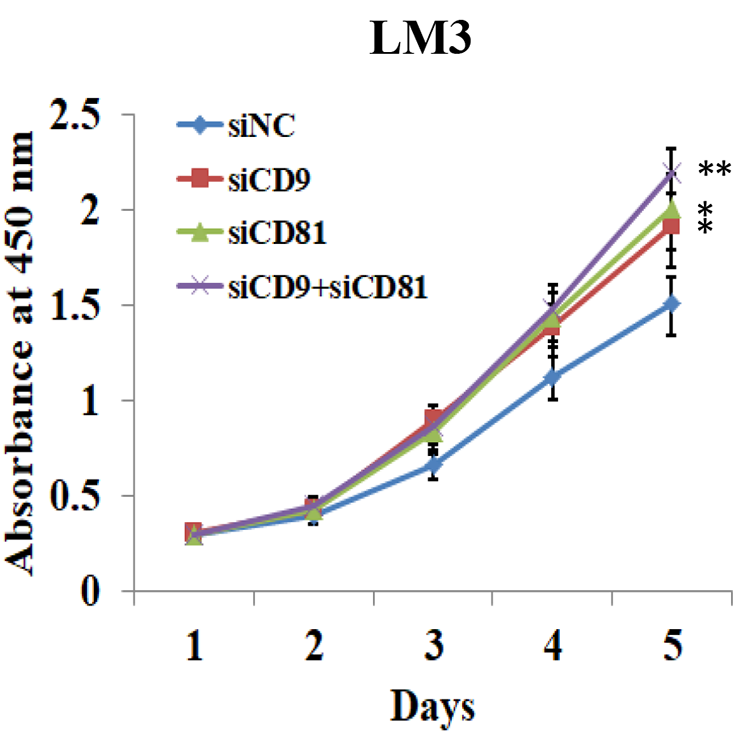

Supplement: Supplementary file 5 — Supplementary Figure 2 [file 41419_2020_2479_MOESM5_ESM.tif]
